# Comparative Toxicity Assessment of Nanosilver on Three *Daphnia* Species in Acute, Chronic and Multi-Generation Experiments

**DOI:** 10.1371/journal.pone.0075026

**Published:** 2013-10-07

**Authors:** Carolin Völker, Cathinka Boedicker, Jan Daubenthaler, Matthias Oetken, Jörg Oehlmann

**Affiliations:** Goethe University Frankfurt am Main, Department Aquatic Ecotoxicology, Frankfurt am Main, Germany; King Abdullah University of Science and Technology, Saudi Arabia

## Abstract

The antibacterial properties of nanosilver have led to a versatile application spectrum including medical purposes and personal care products. However, the increasing use of nanosilver has raised concerns about its environmental impacts. Long-term exposure studies with aquatic invertebrates are essential to assess possible adverse effects on aquatic ecosystems. In the present study, acute (48 h), chronic (21 d) and long-term effects of nanosilver (primary size 15 nm) on five successive generations of three *Daphnia* species (*D. magna*, *D. pulex*, and *D. galeata*) were investigated. Acute EC_50_ values of nanosilver were 121 µg Ag L^−1^ for *D. magna* being the least sensitive species and 8.95 and 13.9 µg Ag L^−1^ for *D. pulex* and *D. galeata*, respectively. Chronic exposure provided EC_10_ values of 0.92 µg Ag L^−1^ for *D. magna* showing the most sensitive chronic reaction and 2.25 and 3.45 µg Ag L^−1^ for *D. pulex* and *D. galeata*, respectively. Comparative exposure to AgNO_3_ revealed a generally higher toxicity of the soluble form of silver. The multi-generation experiments resulted in effects on the population level for all tested species. Exposure of *D. magna* indicated an increased toxicity of nanosilver in the fifth generation of animals exposed to 10 µg Ag L^−1^. Neonates from pre-exposed parental daphnids did not completely recover when transferred into clean water. Exposure of *D. pulex* and *D. galeata* revealed not only increasing toxicity in some generations, but also greater tolerance to nanosilver. This study contributes to the assessment of the risk potential of nanosilver on aquatic ecosystems. It shows that effects of nanosilver vary within one genus and change with exposure duration. Therefore, long-term studies considering different aquatic species are needed to better understand the possible effects of nanosilver on aquatic ecosystems.

## Introduction

Engineered nanomaterials are increasingly produced and utilized in various industrial and commercial sectors. They are applied for their specific characteristics resulting from an altered surface area to volume ratio compared to their bulk counterparts. As the amount and diversity of nanomaterial applications increase, concerns about their release into the environment and their impact on natural ecosystems are growing [1]–[3].

Currently, nanoscale silver is one of the most frequently used nanomaterials in nanotechnology-enabled consumer products [4]. Nanosilver has antimicrobial properties and was initially used for medical purposes and water treatment [5]. The use of nanosilver has meanwhile been extended to a variety of commercially available products, including textiles, cosmetics, food packaging materials and electronics [1]. The use and degradation of these products will likely result in an environmental release of nanosilver [6]. For instance, it has been shown that nanosilver impregnated sock fabrics leach out silver particles [7]. Moreover, the effluent from a silver nanowashing machine was shown to contain silver nanoparticles [8].

Given the high toxicity of silver compounds to aquatic species [9], studies on the environmental impact of silver nanoparticles are imperative. Among nanometals, nanosilver shows most toxic effects on aquatic organisms [10]–[12]. However, the underlying toxic mechanism it is not yet clear [13]. Several studies have demonstrated that release of silver ions from nanoparticle surfaces contribute to nanosilver toxicity [14], [15], while other studies have found different effects of ionic silver (as AgNO_3_) and silver nanoparticles [16], [17].

Among the tested species *Daphnia* responds most sensitively and studies have revealed acute toxic effects in the low microgram per liter range [10], [11], [18], [19]. The low effect values obtained for daphnids are probably due to their filter-feeding strategy leading to an effective uptake of the nanoparticles [11].

Chronic exposure of daphnids to nanosilver results in negative effects on growth and reproduction [20]. However, chronic exposure data are limited and cover exclusively a period of 21 days. To assess the environmental impact of nanosilver, long-term invertebrate exposures giving priority to effects at the population level provide useful completion [21]. A useful approach to predict long-term effects on populations are multi-generation experiments that cover effects on neonate fitness [22], [23].

The aim of this study is to evaluate the effects of nanosilver on consecutive generations of three species of *Daphnia*: *D. magna*, *D. pulex*, and *D. galeata*. We investigated if long-term exposure of several consecutive generations may alter the species’ sensitivity to nanosilver. We further examined how these species differ in their reaction and compared the effects of nanosilver with those exhibited by AgNO_3_.

## Materials and Methods

### 2.1 Test Materials

Polyvinylpyrrolidone- (PVP-) coated nanosilver (NM-300 silver<20 nm) was obtained from RAS GmbH (Regensburg, Germany). Particles were produced by chemical precipitation of AgNO_3_ and show a primary size of 15 nm (TEM). The material is delivered in aqueous dispersion, containing 10% silver, 7% ammonium nitrate, 8% emulsifier, and 75% de-ionised water. NM-300 has been thoroughly characterized by the Joint Research Centre (JRC) of the European Commission [24]. The silver content stated by the manufacturer was confirmed as the JRC determined a mass-related silver content of 9.7±0.4% (ICP-OES).

Correspondingly, as a matrix control, the manufacturer provides the material NM-300 DIS, which contains all dispersant components without silver.

AgNO_3_ used to compare the toxicity of soluble silver components was obtained from Merck KGaA (Darmstadt, Germany).

### 2.2. Preparation and Characterization of Test Materials

Nanosilver dispersions were obtained by preparing a stock dispersion of NM-300. Therefore, 100 mg of NM-300 were added to 20 mL of de-ionised water. To obtain the different exposure concentrations, required amounts of the stock dispersion were pipetted in the test medium. Dispersant controls were prepared in the same manner. Because NM-300 is delivered in dispersion, the amount of the dispersant components varies between the different nanosilver concentrations used in the experiments. To evaluate possible effects of the dispersant, the dispersant proportion of the highest nanosilver concentration was used as a control.

AgNO_3_ solutions were obtained by preparing a stock dispersion of 1 mg AgNO_3_ in 20 mL of de-ionised water. Exposure concentrations were obtained by pipetting required amounts of the stock dispersion in M4 medium.

Nanosilver shapes were determined by transmission electron microscopy. Furthermore, particle size distributions and zeta potential were determined using a zetasizer (Nano ZS90, Malvern Instruments, Worcestershire, UK). Zetasizer analyses were made with nanosilver dispersed in the test medium at concentrations as high as 10 mg L^−1^ in order to ensure an accurate analysis of particle size distributions.

Dissolution of nanosilver was determined by centrifugal ultrafiltration (Millipore Amicon® Ultra 3K) through a cellulose membrane (weight limit 3 kDa). Dispersions were centrifuged for 60 min at 3000 g (Centrifuge 5702, Eppendorf®). The concentration of dissolved silver present in the filtrate was determined by ICP-MS (ELAN DCR-e, Perkin Elmer, Überlingen) and related to the previously inserted concentration.

### 2.3. Test Organisms and Culture Conditions

*D. magna* were obtained from IBACON GmbH (Rossdorf, Germany), *D. pulex*, and *D. galeata* originated from in-house cultures at Goethe-University. Daphnids were cultured in M4 medium [25] at a constant temperature of 20±1°C and a and a photo-period of 16 h light and 8 h dark. Culture medium was renewed twice a week and daphnids were fed a suspension of green algae (*Scenedesmus acutus*).

### 2.4. Multi-generation Experiments

Initially, acute immobilisation tests according to OECD guideline 202 [26] of 48 h duration were conducted to determine the sensitivity range of *D. magna*, *D. pulex*, and *D. gaelata*. Daphnids were exposed to five AgNP and two control (water and dispersant control) treatments. Acute tests were conducted using 20 daphnids divided in four replicates of five animals each.

For the multi-generation experiments, five consecutive generations (F0–F4) of *D. magna*, *D. pulex*, and *D. gaelata* were exposed to nanosilver. Experiments were set up according to Seeland et al. [27], whereby exposure of the respective *Daphnia* generations followed the OECD guideline 211 [28]. One neonate (<24 h old) per glass beaker (100 mL) was exposed to 50 mL M4 medium containing different nanosilver concentrations. 10 replicates per treatment were considered in the study with *D. magna*, 12 replicates in the study with *D. pulex*, and 15 replicates per treatment in the study with *D. galeata*. The exposure concentrations were 2.5, 5, and 10 µg Ag L^−1^ for *D. magna*, 1.25, 2.5, and 5 µg Ag L^−1^ for *D. pulex*, and 1.25, 2.5, 5, and 10 µg Ag L^−1^ for *D. galeata*. In addition, in each experiment a control (only medium) and a dispersant control (medium and NM-300 DIS) were considered.

**Figure 1:**
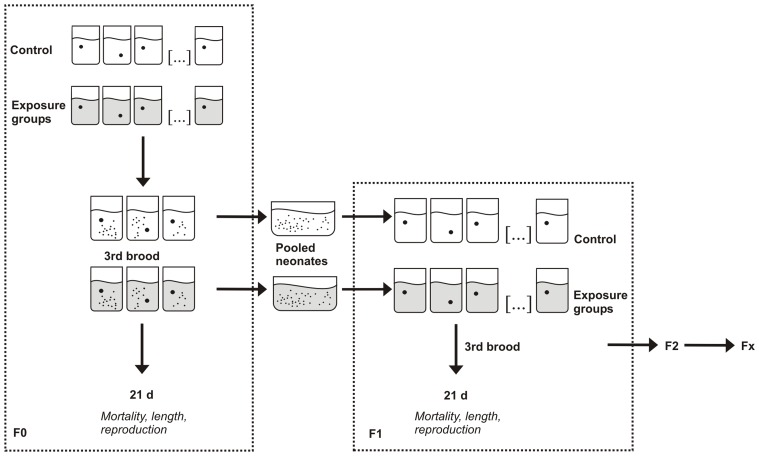
Experimental set-up of the multi-generation study.

To initiate the next generation, the offspring originating from the third brood from at least three different parental animals were pooled and exposed as described before (Fig. 1). If this condition was not met, the population was considered as extinct.

Each generation was exposed for a period of 21 d. During that time, mortality, time of the first brood, and newly born offspring were determined.

The experiments were conducted at a 16∶8 h light:dark cycle and at 20±1°C. To ensure that effects of silver are not altered by the chelating agent EDTA, the M4 medium used in the experiments did not contain EDTA. The medium was changed twice a week and daphnids were fed with green algae *S. acutus* (0.2 mg C daphnid^−1^ d^−1^).

### 2.5. Recovery Experiment

A recovery experiment was conducted with pre-exposed *D. magna* to assess whether daphnids are permanently affected after long-term exposure to silver nanoparticles. Therefore, offspring of previously exposed parental daphnids (F2 of the 10 µg Ag L^−1^ treatment group) was transferred into control medium. According to the design of the multi-generation experiment, the offspring originated from the third brood of the pre-exposed animals. Ten neonates were placed separately in 100 mL glass beakers containing 50 mL of Elendt M4 medium. The daphnids were treated like the control animals for a recovery period of two successive generations and indicated as F3’ and F4’.

### 2.6. Comparative Acute and Chronic Toxicity Tests with AgNO_3_

To test whether effects of nanosilver are comparable to effects exerted by silver ions, we further determined the acute and chronic toxicity of AgNO_3_. *D. pulex* and *D. galeata* were exposed to 0.08, 0.16, 0.32, 0.64, and 1.28 µg Ag L^−1^ and *D. magna* to 0.32, 0.64, 1.27, 2.54, and 5.08 µg Ag L^−1^. The experimental design was chosen as described before. In view of the large scale of the multi-generation experiments, only one generation (21 d) of each species was considered in the tests.

### 2.7. Statistical Analysis

Data were analyzed using GraphPad Prism® (version 5.01, GraphPad Software, San Diego, CA, USA). All data were checked for normal distribution (D’Agostino-Pearson normality test). A two-way ANOVA with Bonferroni post-test was applied to detect significant effects of the parameters time (generation) and toxicity.

The significance level was set at 5% (*p<0.05, **p<0.01, ***p<0.001).

The 10% and 50% effect concentrations were determined using a four-parameter non-linear regression model.

The intrinsic rate of population increase (*r*) was determined based on reproduction and mortality. *R* was calculated iteratively for each treatment and generation according to the Euler-Lotka equation [29].
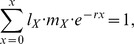
where *x* indicates the age of the individuals, *l_x_* represents the age specific survival and *m_x_* the number of offspring per female released at day *x*.

## Results

### 3.1. Nanoparticle Characterization

**Figure 2:**
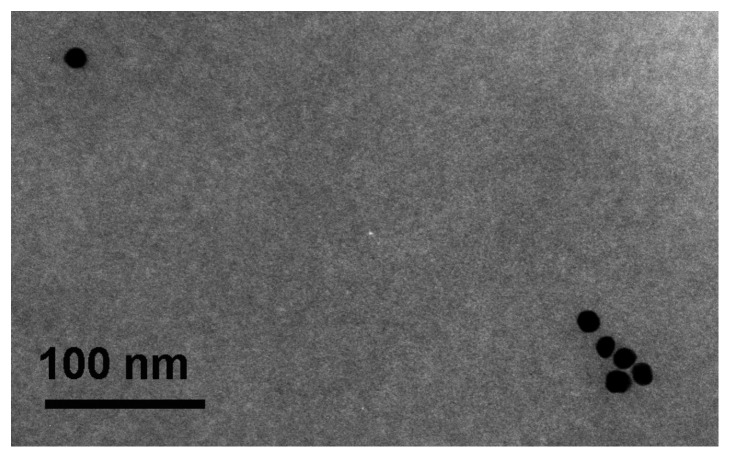
Transmission electron microscopy image of spherical silver nanoparticles (NM-300).

Silver nanoparticles dispersed in M4 medium were spherical in shape as shown by TEM (Fig. 2). DLS measurements revealed a hydrodynamic diameter of 57.6±1.20 nm in the test medium. The zeta potential was −17.0±0.57 mV. The silver nanoparticles showed low dissolution; less than 2% were present in the ionic form after 48 h.

### 3.2. Comparison of Acute and Chronic Effects of Nanosilver and AgNO_3_

AgNO_3_ exhibited a considerably higher acute toxicity compared to nanosilver in all tested *Daphnia* species, although with species-specific differences (Tab. 1). *D. pulex* showed an EC_50_ of 8.95 µg Ag L^−1^ after 48 h exposure to nanosilver, while the EC_50_ for AgNO_3_ was by factor 13 lower (0.68 µg Ag L^−1^). For *D. galeata* the difference was slightly lower with factor 6.5 and an EC_50_ of 13.9 µg Ag L^−1^ for nanosilver and 2.13 µg Ag L^−1^ for AgNO_3_. The greatest difference in acute toxicity was found for *D. magna*. Here the effects of AgNO_3_ were about hundred times higher (EC_50_: 1.10 µg Ag L^−1^) than the effects of nanosilver (EC_50_: 121 µg Ag L^−1^).

**Table 1:** Comparison of effect concentrations of nanosilver and AgNO_3_ on *Daphnia magna*, *Dapnia pulex*, and *Daphnia galeata* after acute exposure (48 h).

		EC_10_ (CI)	EC_50_ (CI)	df	R^2^	SS	Sy.x
*D. magna*	AgNP	60.3 (39.9–91.0)	121 (101–146)	26	0.803	9423	19.04
	AgNO_3_	0.91 (0.84–1.00)	1.10 (1.02–1.21)	26	0.9531	3000	10.74
*D. pulex*	AgNP	4.37 (3.07–6.22)	8.95 (7.54–10.6)	30	0.8779	6000	14.14
	AgNO_3_	0.27 (0.12–0.58)	0.68 (0.48–0.98)	22	0.7045	14832	25.97
*D. galeata*	AgNP	11.0 (8.47–14.3)	13.9 (9.13–21.4)	26	0.9048	3198	11.09
	AgNO_3_	1.71 (1.37–2.12)	2.13 (1.95–2.34)	3	0.9689	217.7	8.518

EC_X_ in µg Ag L^−1^ and CI in brackets.

EC_10_ = concentration causing 10% effect;

EC5_0_ = concentration causing 50% effect;

CI = confidence interval;

AgNP = silver nanoparticles.

However, the consistent pattern of higher toxicity of AgNO_3_ in all species changed in the chronic experiment with *D. magna*. Here we obtained a nearly equal toxicity of nanosilver and AgNO_3_ (EC_10_: 0.92 µg Ag L^−1^ and 0.98 µg Ag L^−1^, respectively) (Tab. 2). Results obtained for *D. pulex* are not directly comparable, because exposure to nanosilver did not affect reproduction. However, the EC_10_ (based on mortality) for nanosilver is 2.25 µg Ag L^−1^ which is about ten times higher than the value obtained for AgNO_3_ (0.16 µg Ag L^−1^). The 21 d chronic exposure of *D. galeata* revealed an EC_10_ value for nanosilver of 3.45 µg Ag L^−1^ and 0.01 µg Ag L^−1^ for AgNO_3_.

**Table 2:** Comparison of effect concentrations of nanosilver and AgNO_3_ on *Daphnia magna*, *Dapnia pulex*, and *Daphnia galeata* after chronic exposure (21 d).

	*D. magna*					*D. pulex*					*D. galeata*				
	EC_10_ (CI)	df	R^2^	SS	Sy.x	EC_10_ (CI)	df	R^2^	SS	Sy.x	EC_10_ (CI)	df	R^2^	SS	Sy.x
AgNP	0.92 (0.33–2.53)	3	0.6246	454.1	12.30	2.25 (0.09–53.8)^1^	2	0.1992	1137	23.84	3.45 (1.07–11.2)	2	0.9599	20.17	3.176
AgNO_3_	0.98 (0.32–3.04)	4	0.6228	314.4	8.866	0.16 (0.03–0.86)	4	0.8050	184.3	6.788	0.01 (0.001–0.46)	4	0.7800	250.2	7.908

EC_10_ in µg Ag L^−1^ based on reproduction, CI in brackets.

1 effect value based on mortality.

### 3.3. Multi-generation Experiments

In the experiments no effects of the NM-300 dispersant were found. For this reason, the dispersant control and the negative control were combined and are presented as ‘control’. All life cycle parameters assessed during the experiment (survival, brood release, offspring number, intrinsic rate of population increase) can be viewed in the Table S1.

#### Mortality

**Figure 3:**
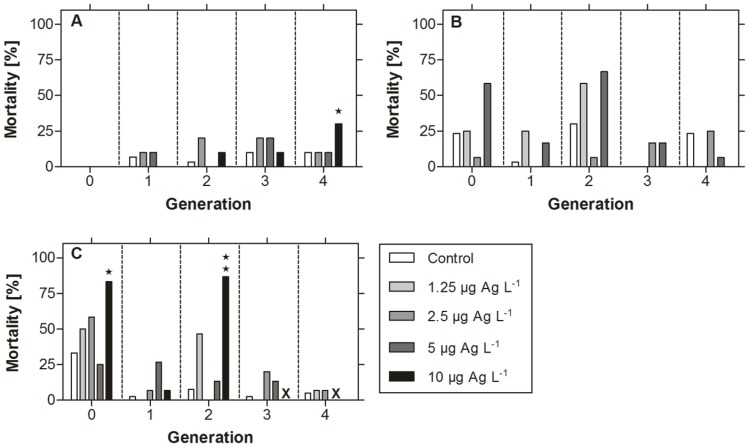
Mortality of daphnids in the multi-generation experiment. Figure shows the mortality of five successive generations of (A) *Daphnia magna*, (B) *Daphnia pulex*, and (C) *Daphnia galeata* exposed to nanosilver. Asterisks indicate significant differences from control: * = p<0.05, ** = p<0.01 (two-way ANOVA and Bonferroni post-test).

*Daphnia magna* showed mortality levels below 20% at all tested concentrations from generation 0 to 3 (Fig. 3A) except at the highest test concentration (10 µg Ag L^−1^) with a mortality of 30.0% in generation 4.

*D. pulex* showed mortality levels between 0 and 30% in generation 0, except for animals exposed to the highest test concentration (5 µg Ag L^−1^), showing 58.3% mortality (Fig. 3B). In generation 1 mortality levels did not exceed 25% for all exposure groups. However, mortality in generation 2 increased in all exposure groups and reached 58.3% (1.25 µg Ag L^−1^) and 66.7% (5 µg Ag L^−1^), respectively. In generation 3 and 4 mortality of all exposure groups ranged between 0 and 25%.

Mortality of *D. galeata* reached up to 83.3% (10 µg Ag L^−1^) in generation 0 (Fig. 3C). In generation 1, the mortality level of the animals exposed to 10 µg L^−1^ decreased to 6.70%, but increased again to 86.7% in the following generation (F2). Given the high mortality level at 10 µg Ag L^−1^ in generation 2, the experiment could not be continued and, therefore, this population was considered as extinct. The remaining treatment groups showed mortality levels between 0 and 27% except for the animals exposed to 1.25 µg Ag L^−1^ in generation 2 (46.7%) while no mortality occurred in generations 3 and 4.

The two-way ANOVA revealed that time contributed significantly to the observed mortality levels of *D. magna* and *D. pulex*, whereas time, toxicity, and the interaction between both parameters contributed to the mortality found for *D. galeata* (Tab. 3).

**Table 3:** Two-way ANOVA testing for the influence of nanosilver toxicity and exposure time on mortality and reproduction of five populations of *Daphnia magna*, *Daphnia pulex*, and *Daphnia galeata*.

	*D. magna*					*D. pulex*					*D. galeata* 1				
	df	SS	MS	F	p	df	SS	MS	F	p	df	SS	MS	F	p
**Mortality**															
Time	4	726.1	181.5	13.75	0.0066	4	3349	837.2	6.739	0.0301	2	4784	2392	57.41	0.0041
Toxicity	3	135.5	45.17	3.422	0.1094	3	1422	473.8	3.814	0.0918	4	4252	1063	25.51	0.0119
Interaction	12	624.2	52.02	3.940	0.0702	12	3716	309.7	2.493	0.1611	8	4167	520.8	12.50	0.0309
**Reproduction**															
Time	4	3044	761	5.005	0.0007	4	27500	6875	36.59	<0.0001	2	7217	3609	63.44	<0.0001
Toxicity	3	31130	1380	68.25	<0.0001	3	701.0	233.7	1.244	0.2946	4	824.9	206.2	3.625	0.0072
Interaction	12	1222	101.8	0.669	0.7800	12	3504	292.0	1.544	0.1063	8	1677	209.6	3.684	0.0005

1 Generations 3 and 4 are not considered because the 10 µg Ag L^−1^ exposure group became extinct.

#### Reproduction

**Figure 4:**
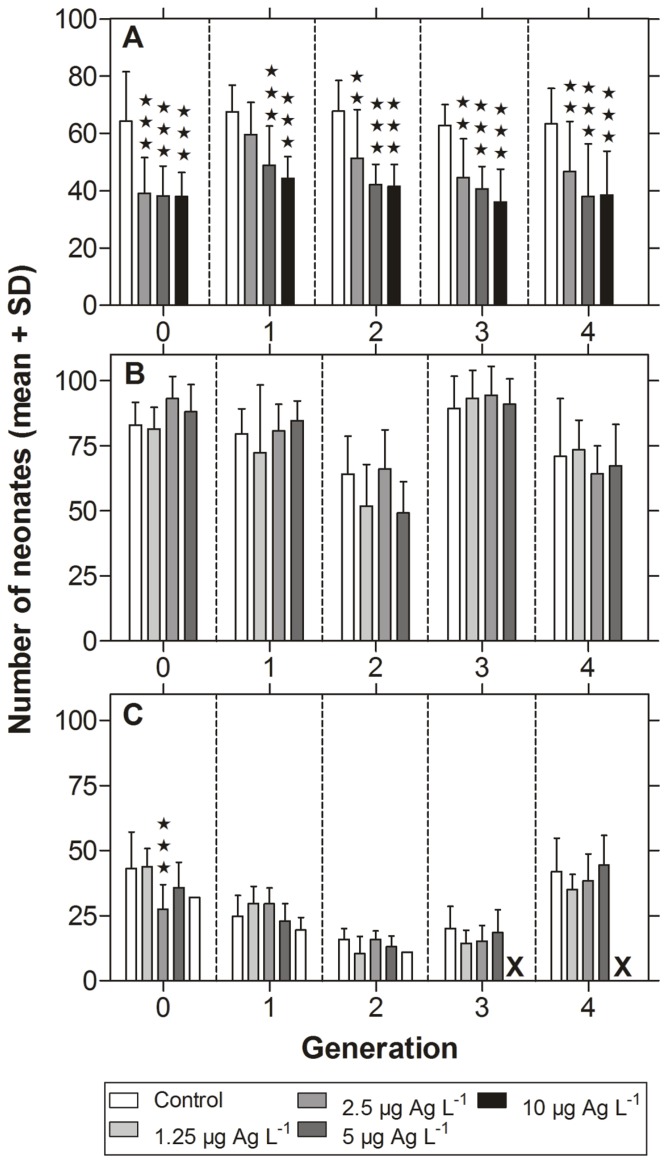
Reproduction of daphnids in the multi-generation experiment. Figure shows the mortality of five successive generations of (A) *Daphnia magna*, (B) *Daphnia pulex*, and (C) *Daphnia galeata* exposed to nanosilver (neonates per adult, mean+SD). Asterisks indicate significant differences from control: ** = p<0.01, *** = p<0.001 (two-way ANOVA and Bonferroni post-test).

Exposure to nanosilver significantly impaired the reproductive output of *D. magna* (Fig. 4A). All tested concentrations resulted in a decreased number of neonates compared to the control group in generation 0. Here the control animals released 64.2 neonates and the exposed animals showed offspring numbers of 39.1, 38.2, and 38.0, at 2.5, 5 and 10 µg Ag L^−1^, respectively. In the subsequent generation (F1), offspring numbers slightly increased in all exposure groups, but reproduction of daphnids treated with 5 and 10 µg Ag L^−1^ was still significantly lower compared to the control. In generations 2–4, reproduction of the different groups remained in an equal range and was significantly and concentration-dependently impaired in all groups treated with nanosilver. The two-way ANOVA indicated that time and toxicity of nanosilver contributed to the effects (Tab. 3).

The reproductive output of *D. pulex* was not affected by low concentrations of nanosilver during the whole experiment (Fig. 4B). Indeed mean neonate numbers varied between generations, but showed comparable levels within one generation. For example, neonate numbers of generation 0 ranged between 81.6 (1.25 µg Ag L^−1^) and 93.2 (2.5 µg Ag L^−1^), whereas neonate numbers of generation 2 ranged between 49.3 (5 µg Ag L^−1^) and 64.2 (control). Results of the two-way ANOVA showed a significant effect of exposure time that contributed to the variations (Tab. 3).

Neonate numbers of *D. galeata* were generally lower compared to the other tested species. In generation 0, the control showed 43.2 neonates (Fig. 4C). Daphnids exposed to 1.25 µg Ag L^−1^ showed slightly higher neonate numbers, whereas daphnids exposed to 2.5 µg Ag L^−1^ showed the lowest neonate numbers (27.4). Nanosilver concentrations of 5 and 10 µg Ag L^−1^ resulted in slightly decreased offspring numbers compared to the control. Generation 1 showed generally lower offspring numbers (24.8 neonates in the control) and neonate production in the exposure groups was in the same range. The same pattern was achieved in generation 2. Although animals exposed to 10 µg Ag L^−1^ showed a high mortality level in this generation, the reproduction did not differ significantly from the control group. Nonetheless, this exposure group had to be considered as extinct due to the high mortality level. In the subsequent generations 3 and 4, the remaining treatment groups showed similar offspring numbers as did the control group. It has to be noted, that reproduction of *D. galeata* in generation 4 increased again to the range of the F0 animals. The two-way ANOVA revealed that both parameters (time and toxicity) as well as their interaction contributed to the observed effects (Tab. 3).

#### Intrinsic rate of population increase

**Figure 5:**
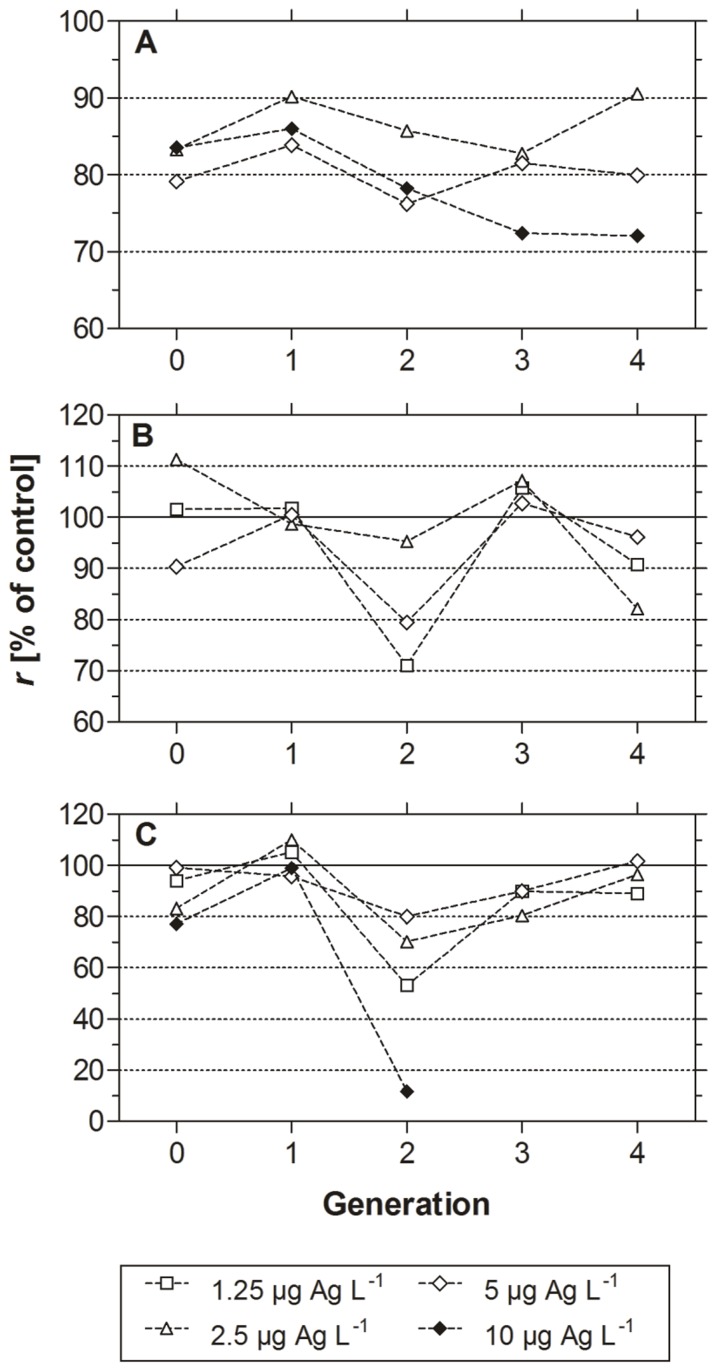
Intrinsic rate of population increase of daphnids in the multi-generation experiment. Figure shows the intrinsic rate of population increase (*r*) of five successive generations of (A) *Daphnia magna*, (B) *Daphnia pulex*, and (C) *Daphnia galeata* exposed to nanosilver (mean % of control). Controls were set to 100% (solid line).

In each generation, the intrinsic rate of population increase (*r*) was determined. In Figure 5 *r* for all species is shown with normalized data relative to the control. Multi-generation exposure of *D. magna* resulted in decreased *r* values at all tested concentrations (Fig. 5A). Animals exposed to 2.5 µg Ag L^−1^ showed values ranging between 83.3 and 90.6% of the control during the whole experiment. The *r* values of daphnids exposed to 5 µg Ag L^−1^ ranged between 79.1 and 83.9% of the control. In contrast to the other groups, the *r* value of the highest exposure group (10 µg Ag L^−1^) decreased during the experiment from 83.5% (F0) to 72.0% (F4).

The *r* values of *D. pulex* were in the same range as the *r* values of the control group in generations 0 and 1 (Fig. 5B). In generation 2, *r* values decreased to 71.0%, 95.2%, and 79.4% of control at 1.25, 2.5, and 5 µg Ag L^−1^, respectively. The *r* values of generation 3 were all in the range of the control group again, whereas *r* values of generation 4 slightly differed (from 82.1% in 2.5 µg Ag L^−1^ to 96.1% in 5 µg Ag L^−1^).

Exposure of *D. galeata* revealed decreased *r* values to 77.1% (10 µg Ag L^−1^) in generation 0 (Fig. 5C). The values for generation 1 were all in the range of the control, whereas *r* values of generation 2 showed the highest deviation from the control group. In this generation, animals exposed to 10 µg Ag L^−1^ only reached 11.7% of the control and were not able to produce a further generation. The *r* values of generations 3 and 4 of nanosilver exposed daphnids gradually increased to the control level again.

### 3.4. Recovery Experiment Over Two Generations

**Figure 6:**
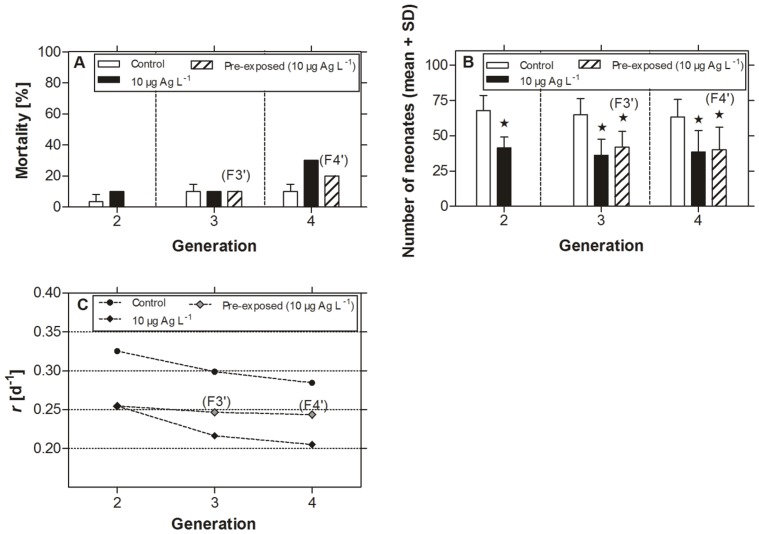
Recovery experiment with *Daphnia magna* after pre-exposure to nanosilver. Figure shows (A) mortality (%), (B) reproduction (neonates per adults, mean+SD), and (C) intrinsic population increase rate (*r*) of the control group, animals still exposed to 10 µg Ag L^−1^, and daphnids in clean water pre-exposed to 10 µg Ag L^−1^. Asterisks indicate significant differences from control: * = p<0.05, (two-way ANOVA and Bonferroni post-test). C = control.

To check whether *D. magna* is permanently damaged due to nanosilver exposure, a recovery experiment was performed. Neonates from previously exposed parental animals (F2 of the 10 µg Ag L^−1^ treatment group) were transferred into clean water for a recovery period of two successive generations. Reproduction of generation F3’ and F4’ was significantly impaired during the recovery period and remained in the range of the 10 µg Ag L^−1^ treatment group of the multi-generation experiment (Fig. 6B). *R* values were slightly decreased compared to the control group but higher than those of the daphnids still exposed to 10 µg Ag L^−1^ (Fig. 6C).

## Discussion

### 4.1 Effects of Particle Chemistry and the Role of Silver Ions

It is well known that particularly soluble silver compounds are highly toxic to aquatic species [30]. It is, however, not clear by which mechanisms silver nanoparticles are toxic. There are divergent opinions on the effects of surface coatings, aggregation state, sizes, and solubility of nanosilver. By now there are some studies that identified acute toxicities of nanosilver on daphnids. For example, Li et al. [10] investigated the effects of different kinds of citrate stabilized silver nanoparticles on *Daphnia magna* and found 48-h LC_50_ values of about 3 µg L^−1^. The particles with primary sizes of 58, 71, and 59 nm agglomerated in the test medium to sizes of 438, 378, and 553 nm, respectively. Although particles of the present study showed less aggregation (57.6 nm), they exhibited a lower toxicity (EC_50_ 121, 8.95, and 13.9 µg Ag L^−1^) than those investigated by Li et al. [10]. The EC_50_ of 121 µg Ag L^−1^ (*D. magna*) is comparable with the mortality level found by Gaiser et al. [31] for *D. magna* (56.7% at 100 µg L^−1^). However, particles used by Gaiser et al. [31] had no surface coating and agglomerated to 588 nm, which is in the range reported by Li et al. [10]. Studies with silver oxide coated silver nanoparticles (peak sizes of 44.5, 94.5, and 216 nm) on adult *D. pulex* and neonates of *Ceriodaphnia dubia* revealed 48-h effect values of 67 and 40 µg L^−1^, respectively. These values are in the range of those obtained in the present study. From these results, no clear classification of the effects exhibited by the particles can be made. The size or the aggregation state of the particles does not allow for definite conclusion on the particles’ toxicity, although other studies found size-dependent toxicities [32].

Zhao and Wang [33] investigated toxicities of particles having different surface coatings, PVP, lactate, and sodium dodecylbenzene sulfonate (SDS). The PVP coated nanoparticles were in an equal size range (79.7 nm) like those used in the present study, but the EC_50_ was much lower (2.0 µg L^−1^). The lactate coated particles showed the largest diameter of 123.9 nm and exhibited the lowest toxicity (28.7 µg L^−1^). Particles coated with SDS had a diameter of 65.3 nm and a LC_50_ of 1.1 µg L^−1^. The authors hypothesized that the different surface coatings influenced the amount of silver ions released from the particle surfaces. Acute toxicity tests with separated silver ions released from the nanoparticles revealed effect concentrations in the range of those obtained for AgNO_3_ [33], [34]. On the contrary, solubility of the silver nanoparticles used in the present study did not reach ion concentrations sufficient to cause acute toxic effects which is in accordance with several other studies [10], [11], [31]. Although other studies indicated that nanosilver elicits higher effects than do silver ions, the results of the present study show higher acute effects of AgNO_3_ (Tab. 1). However, for *D. magna* different results were obtained after chronic exposure to nanosilver and AgNO_3_, respectively (Tab. 2).

In an earlier study, Zhao and Wang [20] investigated the effects of carbonate coated silver nanoparticles (40–50 nm) combined with 1 µM cysteine to complex with silver ions released from the nanoparticles. The authors observed no acute effects on *D. magna* after exposure up to 500 µg L^−1^AgNPs and therefore concluded that toxicity was attributable to solubility. However, it remains unclear if cysteine contributed to the detoxification mechanisms of the daphnids since it is needed for metallothionein synthesis [35].

Furthermore, Zhao and Wang [20] still found chronic sublethal effects of silver nanoparticles even after adding cysteine to complex with released silver ions. Therefore, it remains unclear to which extent the solubility of nanosilver contributes to the toxic effects and further mechanisms may account for nanosilver toxicity. As one possible mechanism, the generation of reactive oxygen species leading to oxidative stress may be attributable to the toxic effects of nanosilver [13].

So far, the toxic mechanism of silver nanoparticles is not yet fully understood. The most important fact, however, remains that silver is one of the most toxic metals to aquatic organisms [9]. Modeled environmental concentrations of nanosilver in surface waters are in the nanogram per liter range [36]. Even though silver nanoparticles are transformed to different silver species in the environment, the increasing use in a variety of consumer products will likely result in higher environmental concentrations of silver compounds. Regarding the fact that silver is a toxic metal, an intensified use of silver nanoparticles may result in detrimental impacts on aquatic ecosystems. More data are needed to fully understand the environmental fate of nanosilver and the effects on aquatic organisms.

### 4.2 Multi-generation Exposure to Silver Nanoparticles

The aim of the multi-generation experiments was to determine whether the toxicity of silver nanoparticles increases over time or whether *Daphnia* populations are able to gain tolerance to silver nanoparticles. Exposure of three species of *Daphnia* to silver nanoparticles over five consecutive generations revealed effects at the population level for all tested species. However, the species exhibited different responses to long-term silver nanoparticle exposure. Population increase of *D. magna* was inhibited at all tested concentration (2.5–10 µg Ag L^−1^) during the multi-generation experiment. There is a trend to an increased toxicity as population growth decreased from 83.5% of control in F0 to 72.0% in F4 (Fig. 5). This effect was mainly due to a higher mortality (Fig. 3) and a delayed time to first brood (Tab. S1).

The multi-generation exposure of *D. pulex* revealed no effects on the population increase rate before generation F2 (Fig. 5) when high mortality levels occurred at 1.25 and 5 µg Ag L^−1^. Interestingly, this effect disappeared in generation F3 and daphnids showed intrinsic rates of population increase that were slightly higher than that of the control. Surviving neonates from generation F2 that were used to initiate the next generation might have been the most tolerant individuals and were therefore not influenced by silver nanoparticle exposure. The multi-generation exposure of *D. galeata* revealed a similar pattern. Daphnids exposed to 10 µg Ag L^−1^ showed a decreased population growth (77.1% of the control group) in the F0 but not in the F1 generation. However, the progeny of the F1 generation did not show any tolerance to silver nanoparticles and became extinct in generation F2.

Although toxicity of metal nanoparticles has not yet been assessed in multi-generation experiments, several multi-generation experiments on the toxicity of metals (in their non-nanoform) exist [22], [37]–[39]. Some studies demonstrated that long-term pre-exposed animals acclimated to the corresponding metal and developed a greater acute tolerance [38], [39]. It is suggested, that this effect might be attributable to the induction of metallothionein-like proteins (MTLP) that prevent organisms from toxic metals [40]–[42]. In the present study, acute toxicity tests were conducted using neonates of each generation pre-exposed to silver nanoparticles, but the 48-h EC_50_ values did not change throughout the whole experiment (data not shown). The more tolerant reaction of some silver exposed generations of *D. pulex* and *D. galeata* might be correlated with the induction of MTLP. However, this detoxification mechanism was not further investigated.

Further multi-generation studies demonstrated reduced chronic tolerance to metals after long-term exposure [37] as indicated in generation F4 in the present study with *D. magna* and in F2 of *D. pulex* and *D. galeata*. Furthermore, recovery experiments with daphnids revealed that neonates of pre-exposed parental daphnids still showed reduced filtration and ingestion rates for a certain time period [43]. In the present study, offspring from pre-exposed *D. magna* did not recover completely even after a period of two generations in clean water (Fig. 6). Several studies pointed out that toxic stress alters energy-reserve fractions (e.g. lipids) in daphnids [37] which are major factors in offspring production [44], [45]. Moreover, the authors of a study on chronic (21 d) effects of nanosilver on *D. magna* hypothesized that negative effects on growth and reproduction resulted from a reduced food intake [20]. The particles were ingested and accumulated in the gut of the daphnids [20], [46]. It turned out that these particles could not be removed easily from the gut and might have inhibited algal food ingestion during the experiment [20]. In the present study, no particles could be observed in the gut of the daphnids, probably due to the low nanoparticle concentrations used in the experiments. Nevertheless, nanoparticles might have altered the food intake and therefore contributed to decreased energy reserves.

**Figure 7:**
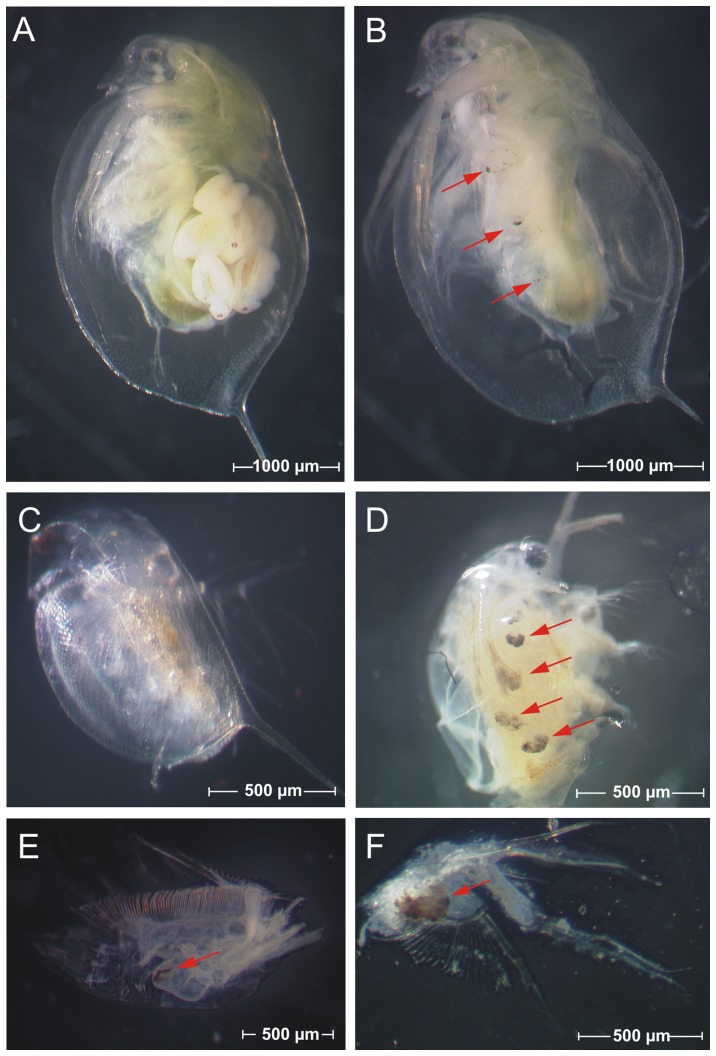
Images of *Daphnia magna*. Figure shows (A) adult individual from the control group, (B) adult individual after acute exposure to nanosilver (20 µg Ag L^−1^), (C) neonate from the control group, (D) neonate after acute exposure to nanosilver, (E-F) dissected trunk limbs of an exposed adult individual. Arrows indicate brown discolorations in the area of the gills after exposure to nanosilver.

Although no silver nanoparticles could be observed in the gut, acute exposure to 20 Ag µg nanosilver L^−1^ revealed brown discolorations of the gill area located on the trunk limbs that were not evident in the control group (Fig. 7). Similar observations were made by Gaiser et al. [31] after treating *D. magna* with 100 µg L^−1^ silver nanoparticles for 96 h. It cannot be finally determined if the brown discolorations observed in the present study are silver nanoparticles that bound to or accumulated in the gills. However, it is remarkable that these discolorations only appeared after treatment with silver nanoparticles at concentrations of 20 µg Ag L^−1^ or higher.

### 4.3 Species-specific Reactions to Silver Nanoparticles

*D. magna* appeared to be the least sensitive species during acute exposure (EC_50_: 121 µg Ag L^−1^), while the population increase was lower at all tested concentration (2.5–10 µg Ag L^−1^) during the multi-generation experiment.

*D. pulex* reacted most sensitively during acute exposure (EC_50_: 8.95 µg Ag L^−1^). Interestingly, no effects on sublethal endpoints could be detected in generations F0 and F1 of the multi-generation study. Although the population increase rate was affected in generation F2, this effect is attributable to high mortality levels of the animals since reproduction was not significantly affected by nanosilver.

*D. galeata*, the smallest of the investigated *Daphnia* species, showed an acute EC_50_ of 13.9 µg Ag L^−1^. The greatest inhibition of the population increase rate during the multi-generation experiment was observed in F2 leading to an extinction of the population exposed to the highest test concentration (10 µg Ag L^−1^). Similar to the experiment with *D. pulex*, reproduction of *D. galeata* was less affected by silver nanoparticles, instead mortality levels increased.

The lower acute sensitivity of *D. magna* might be due to its larger size compared to the other species as indicated in studies comparing the susceptibilities of different *Daphnia* species [47]. The larger surface area to volume ratio of smaller species might lead to higher accumulation rates of the particles and therefore cause lower toxicity thresholds compared to larger species [47]. However, these observations cannot be generalized since other studies could not identify differences in sensitivities of *Daphnia* species [48], [49].

As described previously, Zhao and Wang [20] pointed out that chronic effects of silver nanoparticles were possibly a result of feeding depression due to accumulation of particles in the digestive tract of the daphnids. According to this hypothesis, particles might have been differently ingested by the species due to differences in their filtering activity and morphology of the filtering apparatus [50], [51].

In general, the present study revealed that reactions to silver nanoparticles can vary widely within one genus. An adequate risk assessment of nanosilver should therefore include toxicity data of as many different species as possible.

## Conclusion

In the present study, effects of nanosilver have been assessed in multi-generation experiments for the first time. Exposure of *D. magna*, *D. pulex*, and *D. galeata* demonstrated that effects of nanosilver varied within one genus and the species’ sensitivity changed with exposure duration. The multi-generation experiments indicated an increasing toxicity of nanosilver over time and are therefore useful to better understand possible hazardous effects of nanosilver on aquatic ecosystems.

## Supporting Information

Table S1 Life cycle parameters of five generations of *Daphnia magna*, *Daphnia pulex*, and *Daphnia galeata* after long-term exposure to nanosilver.
(DOCX)
