# Supplementary material for: Comparative Toxicity Assessment of Nanosilver on Three Daphnia Species in Acute, Chronic and Multi-Generation Experiments
Source: PLoS One. 2013 Oct 7;8(10):e75026. doi: 10.1371/journal.pone.0075026 (PMC3792065; doi:10.1371/journal.pone.0075026)
Supplement: Table S1 — Life cycle parameters of five generations of Daphnia magna, Daphnia pulex, and Daphnia galeata after long-term exposure to nanosilver. (DOCX) [file pone.0075026.s001.docx]

|  | |  | Survival [%] | | | | |  | Brood release [d] | | | | |  | Total offspring number | | | | |  | Intrinsic rate of population increase d^-1^ | | | | |
| --- | --- | --- | --- | --- | --- | --- | --- | --- | --- | --- | --- | --- | --- | --- | --- | --- | --- | --- | --- | --- | --- | --- | --- | --- | --- |
| Test conc. [µg L^-1^] | |  | C | 1.25 | 2.5 | 5 | 10 |  | C | 1.25 | 2.5 | 5 | 10 |  | C | 1.25 | 2.5 | 5 | 10 |  | C | 1.25 | 2.5 | 5 | 10 |
|  | F0 |  | 100 |  | 100 | 100 | 100 |  | 9.40 |  | 11.4 | 11.2 | 10.4 |  | 64.2 |  | 39.1 | 38.2 | 38.0 |  | 0.30 |  | 0.25 | 0.24 | 0.25 |
|  | F1 |  | 93.3 |  | 90.0 | 90.0 | 100 |  | 8.61 |  | 9.78 | 10.7 | 9.89 |  | 67.8 |  | 59.6 | 48.9 | 44.3 |  | 0.33 |  | 0.30 | 0.28 | 0.28 |
| *D. magna* | F2 |  | 96.7 |  | 80.0 | 100 | 90.0 |  | 9.03 |  | 11.3 | 11.4 | 11.1 |  | 67.8 |  | 51.4 | 42.1 | 41.4 |  | 0.33 |  | 0.28 | 0.25 | 0.25 |
|  | F3 |  | 90.0 |  | 80.0 | 80.0 | 90.0 |  | 10.3 |  | 11.3 | 11.9 | 12.0 |  | 64.8 |  | 44.6 | 40.6 | 36.1 |  | 0.30 |  | 0.25 | 0.24 | 0.22 |
|  | F4 |  | 90.0 |  | 90.0 | 90.0 | 70.0 |  | 9.67 |  | 11.4 | 13.0 | 14.2 |  | 63.3 |  | 46.7 | 38.1 | 38.5 |  | 0.29 |  | 0.26 | 0.23 | 0.20 |
|  | F0 |  | 76.7 | 75.0 | 91.7 | 41.7 |  |  | 7.61 | 7.33 | 7.46 | 7.60 |  |  | 83.0 | 81.6 | 93.2 | 88.2 |  |  | 0.34 | 0.35 | 0.38 | 0.31 |  |
|  | F1 |  | 96.7 | 75.0 | 100 | 83.3 |  |  | 7.55 | 7.11 | 7.67 | 7.90 |  |  | 72.7 | 72.4 | 80.7 | 84.7 |  |  | 0.35 | 0.35 | 0.34 | 0.35 |  |
| *D. pulex* | F2 |  | 70.0 | 41.7 | 91.7 | 33.3 |  |  | 8.52 | 11.8 | 11.1 | 10.8 |  |  | 64.2 | 51.8 | 66.1 | 49.3 |  |  | 0.28 | 0.19 | 0.27 | 0.22 |  |
|  | F3 |  | 100 | 100 | 83.3 | 83.3 |  |  | 9.13 | 7.75 | 7.60 | 8.80 |  |  | 89.4 | 93.3 | 94.5 | 90.9 |  |  | 0.35 | 0.37 | 0.38 | 0.36 |  |
|  | F4 |  | 76.7 | 100 | 75.0 | 91.7 |  |  | 7.22 | 7.75 | 8.33 | 7.82 |  |  | 71.0 | 73.5 | 64.2 | 67.4 |  |  | 0.38 | 0.34 | 0.31 | 0.36 |  |
|  | F0 |  | 66.7 | 50.0 | 41.7 | 75.0 | 16.7 |  | 9.77 | 9.38 | 10.4 | 9.91 | 10.2 |  | 43.2 | 43.8 | 27.4 | 35.8 | 32.0 |  | 0.24 | 0.23 | 0.20 | 0.24 | 0.19 |
|  | F1 |  | 97.5 | 100 | 93.3 | 73.3 | 93.3 |  | 12.6 | 11.7 | 10.2 | 11.5 | 11.2 |  | 24.8 | 29.6 | 29.5 | 22.9 | 19.5 |  | 0.20 | 0.21 | 0.22 | 0.19 | 0.19 |
| *D. galeata* | F2 |  | 92.5 | 53.3 | 100 | 86.7 | 13.3 |  | 12.0 | 14.8 | 12.8 | 12.6 | 11.0 |  | 15.8 | 10.4 | 15.9 | 13.1 | 11.0 |  | 0.19 | 0.10 | 0.13 | 0.15 | 0.02 |
|  | F3 |  | 97.5 | 100 | 80.0 | 86.7 | -- |  | 12.6 | 11.4 | 14.2 | 13.8 | -- |  | 20.0 | 14.3 | 15.3 | 18.4 | -- |  | 0.18 | 0.16 | 0.14 | 0.16 | -- |
|  | F4 |  | 95.0 | 93.3 | 93.3 | 100 | -- |  | 9.93 | 11.0 | 9.43 | 9.53 | -- |  | 41.8 | 35.0 | 38.4 | 44.4 | -- |  | 0.25 | 0.22 | 0.24 | 0.25 | -- |
